# Comparative Proteomic Analysis of the Mesenchymal Stem Cells Secretome from Adipose, Bone Marrow, Placenta and Wharton’s Jelly

**DOI:** 10.3390/ijms22020845

**Published:** 2021-01-15

**Authors:** Sungho Shin, Jeongmin Lee, Yumi Kwon, Kang-Sik Park, Jae-Hoon Jeong, Suk-Joo Choi, Sa Ik Bang, Jong Wook Chang, Cheolju Lee

**Affiliations:** 1 Center for Theragnosis, Korea Institute of Science and Technology, Seoul 02792, Korea; sungho@kist.re.kr (S.S.); ymkwon@kist.re.kr (Y.K.); 2 KHU-KIST Department of Converging Science and Technology, Kyung Hee University, Seoul 02447, Korea; kspark@khu.ac.kr; 3 Stem Cell & Regenerative Medicine Institute, Samsung Medical Center, Seoul 06351, Korea; wannabeasky@gmail.com; 4 R&D Center, ENCell Co., Ltd., Seoul 06351, Korea; 5 Department of Physiology, School of Medicine, Kyung Hee University, Seoul 02447, Korea; 6 Division of Radiation Cancer Research, Korea Institute of Radiological and Medical Sciences, Seoul 01812, Korea; jeongj@kirams.re.kr; 7 Department of Obstetrics and Gynecology, Samsung Medical Center, Sungkyunkwan University School of Medicine, Seoul 06351, Korea; drmaxmix.choi@samsung.com; 8 Department of Plastic Surgery, Samsung Medical Center, Sungkyunkwan University School of Medicine, Seoul 06351, Korea; si55.bang@samsung.com; 9 Division of Bio-Medical Science & Technology, KIST School, Korea University of Science and Technology, Seoul 02792, Korea

**Keywords:** mesenchymal stem cells, secretome, adipose, bone marrow, placenta, Wharton’s jelly, mass spectrometry

## Abstract

Mesenchymal stem cells (MSCs) have the potential to be a viable therapy against various diseases due to their paracrine effects, such as secretion of immunomodulatory, trophic and protective factors. These cells are known to be distributed within various organs and tissues. Although they possess the same characteristics, MSCs from different sources are believed to have different secretion potentials and patterns, which may influence their therapeutic effects in disease environments. We characterized the protein secretome of adipose (AD), bone marrow (BM), placenta (PL), and Wharton’s jelly (WJ)-derived human MSCs by using conditioned media and analyzing the secretome by mass spectrometry and follow-up bioinformatics. Each MSC secretome profile had distinct characteristics depending on the source. However, the functional analyses of the secretome from different sources showed that they share similar characteristics, such as cell migration and negative regulation of programmed cell death, even though differences in the composition of the secretome exist. This study shows that the secretome of fetal-derived MSCs, such as PL and WJ, had a more diverse composition than that of AD and BM-derived MSCs, and it was assumed that their therapeutic potential was greater because of these properties.

## 1. Introduction

In early studies, mesenchymal stem cells (MSCs) [1] were first associated with the potential for differentiation into specific cells [2,3,4,5,6]. While many studies have described the self-renewal and differentiation properties of MSCs as progenitors, recent studies are more focused on the non-progenitor functions of MSCs, such as immune modulation and the secretion of trophic and protective factors (paracrine actions) [7,8]. Based on the clinical safety of the immunomodulatory feature, the applications of MSCs in clinical trials are diverse: bone and cartilage diseases, neurological diseases, cardiovascular diseases, graft-versus-host disease (GVHD), diabetes, hematological diseases, inflammatory diseases, and diseases in liver and kidneys [9,10].

One of the unique characteristics of MSCs is that they can be isolated from various origins [11,12]. MSCs are known to exist in numerous perivascular tissues (including bone marrow (BM), adipose tissue (AD), peripheral blood, teeth, skeletal muscle, placenta (PL), umbilical cord, amniotic fluid and cord blood), while other stem cells are distributed mainly in the tissues of their defined lineage. Many comparison studies of source-specific MSCs [13,14,15,16] have shown that these cells share common characteristics, and these have been proposed by the International Society for Cell Therapy [17].

**Figure 1:**
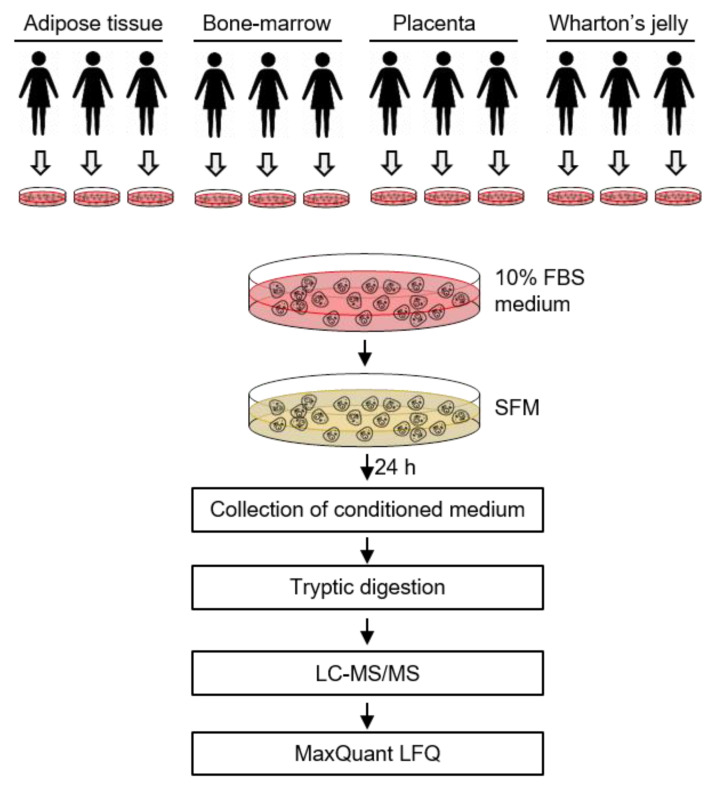
Analysis of the secretome from mesenchymal stem cells (MSCs). MSCs isolated from adipose tissue (*n* = 3), placenta (*n* = 3) and Wharton’s jelly (*n* = 3) were incubated in serum-free conditioned media for 24 h. The conditioned media were collected, concentrated and analyzed by LC–MS/MS.

The expression of genes and proteins are known to have an important role in tissue specificity [18,19]. Thus, the origin of MSCs may contribute to the protein expression, including those found in the secretome. In this study, we compared the secreted proteins in the culture media of human MSCs derived from four different sources: AD, BM, PL, and Wharton’s jelly (WJ) (Figure 1). For this purpose, we used label-free quantification (LFQ) mass spectrometry (MS) because it allows the relative comparison of proteomes between samples, showing the proteins that are released more often from certain cell types [20]. Our results confirmed a significant difference in the secretome profile between MSCs depending on each source. However, the functional analyses of the secretome from different MSC sources were similar despite variation in the composition of the secretome.

## 2. Results

### 2.1. Characterization of MSCs from Different Sources

**Figure 2:**
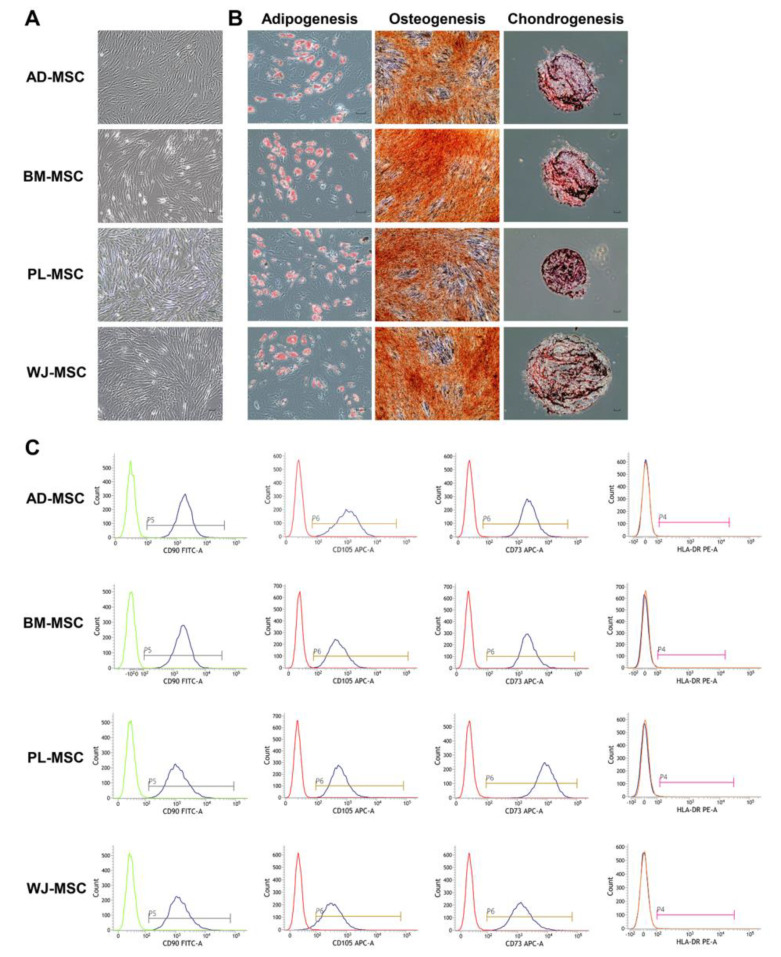
Stemness validation of mesenchymal stem cells (MSCs). MSCs were characterized according to the criteria suggested by the International Society for Cell and Gene Therapy. (**A**) Plastic-adherent features of MSCs are shown. (scale bar = 100 μm) (**B**) Differentiation potentials of MSCs into adipocytes (Oil red O, scale bar = 150 μm), osteocytes (Alizarin Red S, scale bar = 150 μm), and chondrocytes (Safranin O, scale bar = 100 μm) are shown. (**C**) Cell surface markers of MSCs were identified using flow cytometry. Positive markers (CD90, CD105, and CD73) and a negative marker (HLA-DR) are shown. AD-MSC, adipose-derived MSC; BM-MSC, bone marrow-derived MSC; PL-MSC, placenta-derived MSC; WJ-MSC, Wharton’s jelly-derived MSC.

Human MSCs that were isolated from four different sources (AD, BM, PL, and WJ) adhered to plastic cell culture plates and exhibited a spindle-shape (Figure 2A). The MSCs revealed the potential to differentiate into a mesodermal lineage. Mineralization of the extracellular matrix and accumulation of lipid-rich vacuoles inside the cells were detected as a result of osteogenesis and adipogenesis. Chondrogenesis was verified by the expression of sulfated proteoglycans (Figure 2B). Surface marker analyses of MSCs were all positive for CD73, CD90 and CD105, and negative for CD11b, CD19, CD34, CD45 and HLA-DR (Figure 2C and Figure S1). The percentage of positive markers were more than 90%, with negative markers of less than 1%.

### 2.2. Profiling of MSCs Secretome by Mass Spectrometry

**Figure 3:**
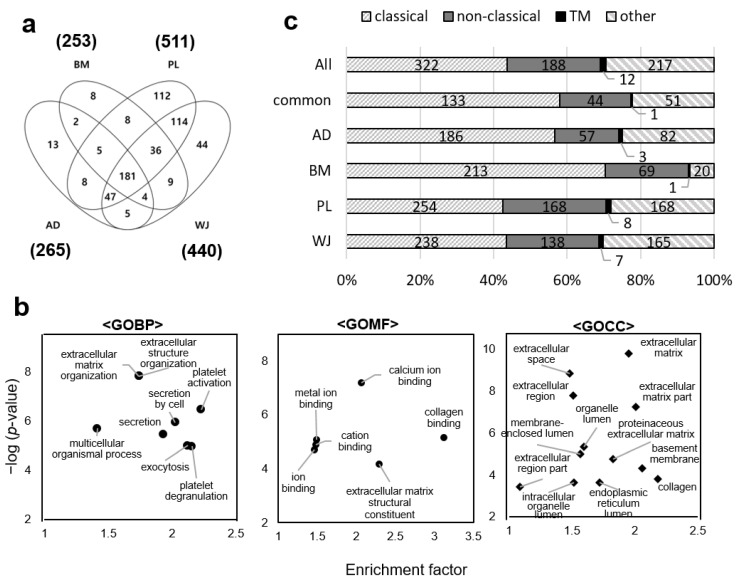
Gene ontology and secretion pathway analysis of mesenchymal stem cell (MSC) secretomes. (**a**) Venn diagram of the secretomes from adipose (AD), bone marrow (BM), placenta (PL), and Wharton’s-jelly (WJ)-derived MSCs. Proteins identified in at least two donors out of three were considered. (**b**) Gene ontology terms enriched by the 181 proteins commonly identified in all MSCs. Gene ontology (GO) terms of biological process (BP), molecular function (MF) and cellular component (CC) are shown. The analysis was performed using Perseus (*p*-value < 0.01). (**c**) Type of secretory pathways predicted by using bioinformatics tools SignalP, SecretomeP and TMHMM. All: proteins identified in any of the four MSCs. Common: 181 proteins identified commonly in all four types of MSCs.

Secreted proteins from MSCs from different sources were analyzed. We analyzed each secretome in a single LC–MS/MS run and quantified protein abundance by using the MaxLFQ algorithm to compare patterns of protein profiles between donors and sources. As a result, we obtained about 13,700 ± 600 peptide spectrum matches (PSMs) for AD-MSCs, 18,000 ± 400 PSMs for BM-MSCs, 19,200 ± 700 PSMs for PL-MSCs and 14,900 ± 400 PSMs for WJ-MSCs. We assessed the within-type variations at the peptide level by comparing the identified peptides between samples of three donors from each MSC source. More than 45% of peptides were identified in all three samples, and more than 67% were identified in at least two samples (Figure S2). When the peptides were compared between different sources of MSCs, the proportion of the peptides identified in all sources was 25%. We excluded proteins that matched the bovine amino acid sequence when sorting MS data at the protein level. In addition, the proteins identified in at least two donors for each source of MSCs were only considered for precise quantification (Figure S3). As a consequence, we identified a total of 596 human proteins: 265 proteins in AD-MSCs, 253 in BM-MSCs, 511 in PL-MSCs and 440 in WJ-MSCs. (Figure 3a and Table S1).

### 2.3. Gene Ontology Analysis of MSCs Secretome and Clustering of Differential Expressed Proteins

Gene ontology (GO) enrichment analysis was performed on 181 proteins identified in all of the MSC sources by a Fisher’s exact test. GO enriched terms in the biological process were extracellular matrix (ECM) organization, platelet activation, exocytosis and secretion. The molecular functions of the proteins were ion binding, collagen-binding and ECM structural constituent. Cellular localization of the proteins was predicted to be the ECM, organelle lumen and vesicles (Figure 3b).

The proteins from each source of MSCs were further analyzed by using in silico secretion pathway prediction programs SignalP, SecretomeP and TMHMM because the GO terms enriched by the commonly identified proteins in MSCs were all related to secretion. About 78% of 181 common proteins were predicted to be truly secretory (Figure 3c). Focusing on each source of MSCs, 75% and 80% of the proteins identified in the AD-MSC and BM-MSC secretomes, respectively, were predicted as secretory; of these, 57% and 60% were predicted to contain signal peptides and were thus secreted through the classical secretory pathway. In the PL-MSCs and WJ-MSCs, 69% and 70% of the proteins were predicted to be secretory, respectively, and proteins predicted to have a signal peptide accounted for about 41% and 43% of the total.

**Figure 4:**
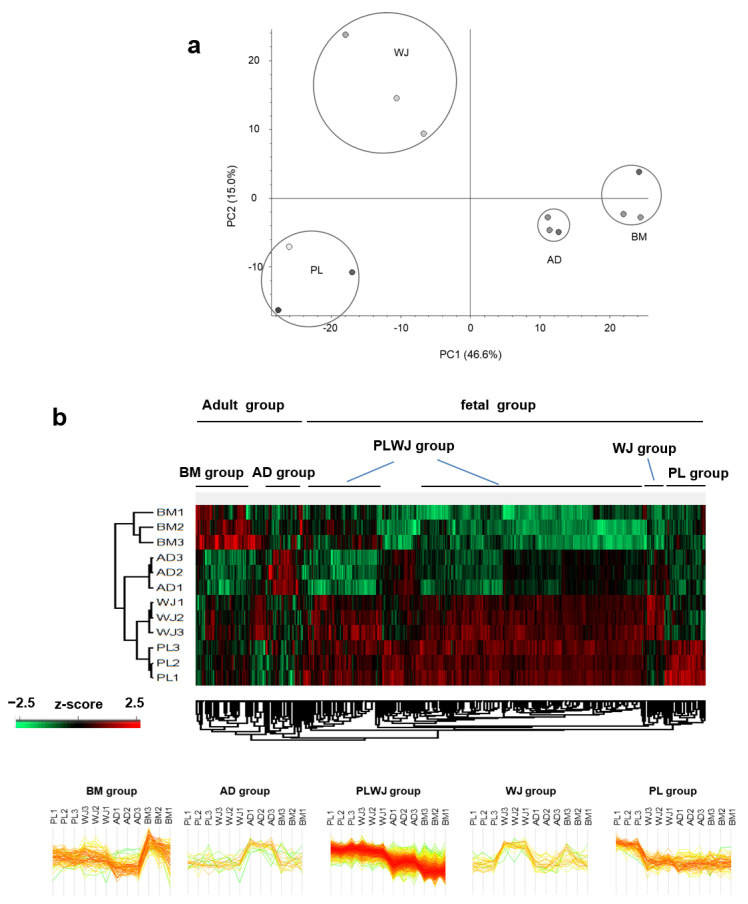
Classification of mesenchymal stem cells (MSCs) according to secretome profile by source. (**a**) Principal component analysis of four mesenchymal stem cell (MSC) secretomes using Ingenuity Pathway Analysis. (**b**) Unsupervised hierarchical clustering of MSC secretome profiles using Perseus. LFQ intensity data were used for the clustering. Proteins that are highly released from a specific MSC source are designated as group proteins of that source. Note that the secretomes from the same source type are clustered more closely to each other.

The difference in the expression patterns of secreted proteins was compared between donors and between MSC sources. Principal component analysis of LFQ results showed that secretome profiling was classified according to the source (Figure 4a). For this, LFQ intensities of MS data were normalized by z-score and analyzed through unsupervised hierarchical clustering (Figure 4b). Secretome from the same source was clustered together, showing that changes in protein expression patterns were more significant between different sources than donor variations.

The secretome differences could be categorized into five major groups: “BM group”, highly secreted proteins in BM-MSCs; “AD group”, highly secreted proteins in AD-MSCs; “PL group”, highly secreted proteins in PL-MSCs; “WJ group”, highly secreted proteins in WJ-MSCs; and “PLWJ group”, highly secreted proteins in PL and WJ-MSCs (Table S2). In addition, WJ-MSCs and PL-MSCs were more closely related than other MSCs in terms of secretome profile, which was also verified by unsupervised hierarchical clustering.

### 2.4. Functional Analyses of Secretome from Different Sources of MSCs

In an analysis using Ingenuity Pathway Analysis (IPA) software and GO, the classification of groups based on protein abundance was expected to show different biological functions. In the AD, PL, WJ and PLWJ groups, but not in the BM group, proteins involved in cellular migration and reduction of apoptosis were abundant in the secretome, although the level differed among the sources (Figure S4). There was no significant IPA result in the BM group since very few proteins were functionally related when compared with other MSCs sources.

The characteristics of the AD group were confirmed as migration and anti-apoptosis. The biological properties of BM-MSCs were identified as the same. Since the functional characterization of secreted proteins from AD and BM-MSCs could not be distinguished, we classified AD and BM-MSCs as adult MSCs, while PL and WJ-MSCs were classified as fetal MSCs. Adult MSCs and fetal MSCs were analyzed separately.

**Figure 5:**
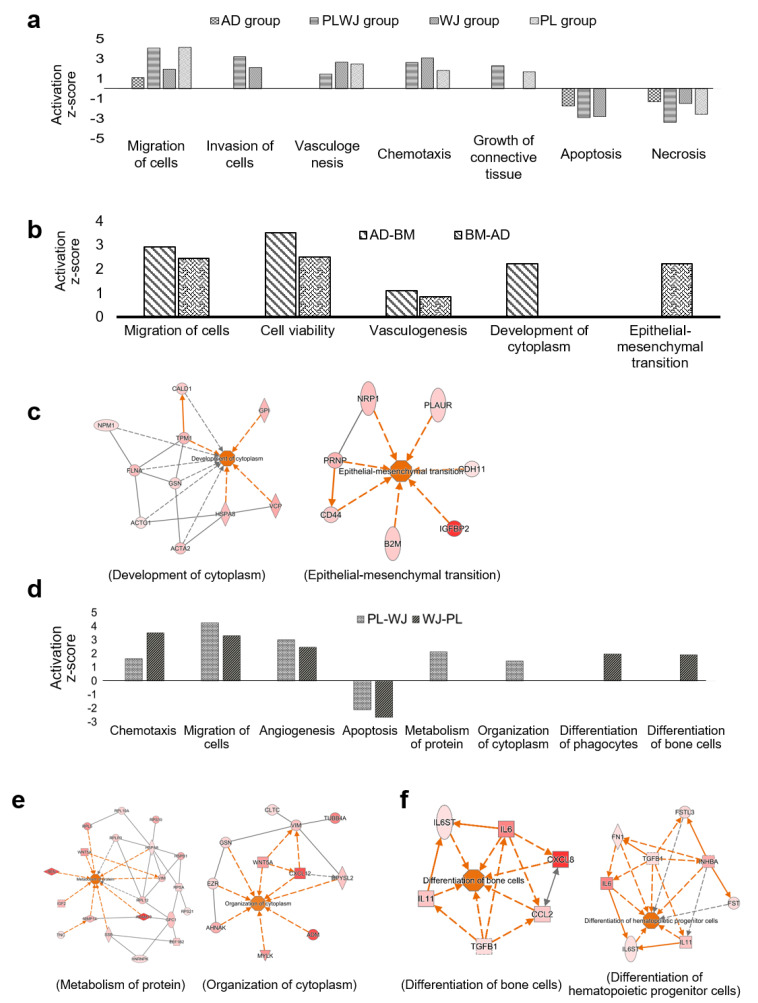
Functional characterization of mesenchymal stem cell (MSC) secretomes derived from different sources. (**a**) Prediction of biological functions (*p*-value < 0.01) of group-specific proteins as designated in Figure 4b. (**b**) Comparison of functional characteristics of BM and AD-MSCs secretomes, which are adult-derived MSCs (*p*-value < 0.01). AD-BM indicates biological functions enriched by the proteins that increased two-fold more in AD-MSCs than in BM-MSCs, while BM-AD indicates the opposite, the biological functions enriched by the decreased proteins in AD-MSCs. (**c**) Protein–protein interaction (PPI) networks are represented by the biological functions that appear only in AD-BM (development of cytoplasm) and BM-AD (epithelial-mesenchymal transition) with activation z-score > 1.5. (**d**) Comparison of functional characteristics between PL and WJ-MSC secretome, that are fetal-derived MSCs (*p*-value < 0.01). PL-WJ: the proteins increased two-fold more in PL-MSCs than in WJ-MSCs; WJ-PL, the proteins increased two-fold more in WJ-MSCs than in PL-MSCs. (**e**,**f**) PPI networks related to the biological functions of PL-WJ (**e**) and (**f**) WJ-PL (activation z-score > 1.5). All of the above analyses were performed using the Ingenuity Pathway Analysis program.

IPA was performed with increased proteins in adult MSCs (Figure 5b). Proteins related to the migration of cells and cell survival were detected commonly (Figure S5). AD-MSCs had more proteins related to the organization, such as the development of cytoplasm, while BM-MSCs had more proteins related to cellular development, epithelial-mesenchymal transition (EMT) and chemotaxis (Figure 5c).

The IPA results of increased proteins in fetal MSCs are also shown. The proportion of proteins that play a role in angiogenesis, another major therapeutic function of MSCs, appeared to be similar (Figure 5d). The secretome of PL-MSCs is expected to show a strong ability for the organization of the cytoplasm and protein synthesis (Figure 5e and Figure S6). On the other hand, more proteins related to tissue development and differentiation of hematopoietic cells were in the secretome of WJ-MSCs (Figure 5f and Figure S6). All groups were expected to increase the migration and invasion of cells and were also predicted to increase the survival of cells. Nevertheless, the secretome of fetal MSCs contains more proteins than adult MSCs. Thus, the groups from PL and WJ-MSCs were expected to show higher potential (Figure 5a).

**Figure 6:**
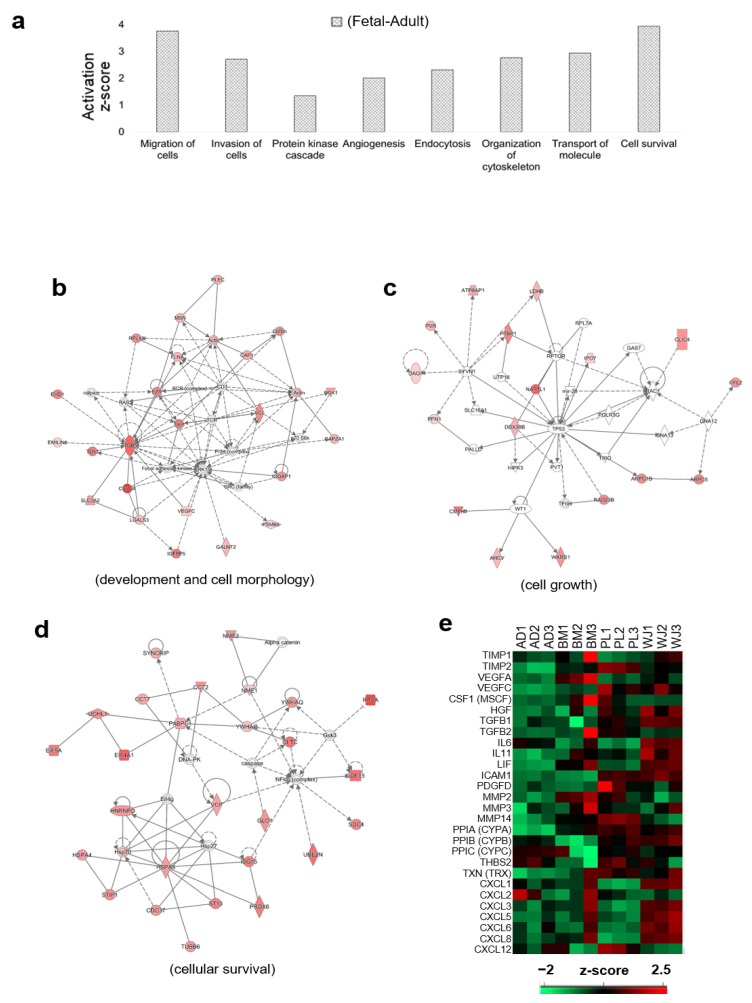
Functional characteristics of fetal-derived mesenchymal stem cell (MSC) secretomes. (**a**) Biological functions of the proteins more abundant in the secretome of PL and WJ-MSCs (Fetal MSCs) than in the AD and BM-MSCs (adult MSCs) (*p*-value < 0.01). (**b**–**d**) Protein networks with the highest enrichment scores for each of three functional terms related to the therapeutic capacity of MSCs—development and cell morphology (**b**), cell cycle and cell growth (**c**), and cell survival (**d**). The network analysis was performed with the Ingenuity Pathway Analysis program. (**e**) Abundance profile of the proteins that are known to have therapeutic abilities in MSCs.

By comparing the functional aspects of the secretome from fetal and adult MSCs, we further analyzed the secretome characteristics of fetal MSCs (Figure 6a). In the secretome of fetal MSCs, more proteins formed a network related to development, cell movement, cell survival, and cellular function. Protein interaction networks related to cell morphology, cellular movement, cellular assembly, cellular organization and embryonic development were found (Figure 6b and Figure S7). There were many proteins involved in tissue differentiation through ITGB-ERK1/2 pathways. It was also speculated that there was a TP53-Rac1 pathway related to cell growth and Hsp70 and NFkB pathways related to cell death and survival.

The therapeutic capacity of MSCs was compared with the expression levels of major proteins (Figure 6c). TIMP, VEGF, CSF, HGF, TGFB, IL6 and PDGF, related to angiogenesis, appeared to be in most of the PL or WJ secretomes [21,22,23,24,25,26]. MMP proteins related to tissue development and remodeling were abundant in the BM and PL secretomes [27], and proteins related to antiapoptotic activity, PPIA, PPIB, and PPIC [28,29], were identified in AD, WJ and PL. Along with the cytokine and growth factors IL-6, IL-11, LIF and ICAM, the cytokine ligand (CXCL) was mainly found in the secretome of WJ-MSCs [23,30,31,32].

## 3. Discussion

MSCs have multi-potential to differentiate into mesodermal tissues. Moreover, these cells secrete trophic and immunomodulatory proteins, thus establishing a regenerative microenvironment [7]. The ability to regulate immune responses and promote the survival of damaged cells is considered to be a key benefit of the application of MSCs as therapeutics. With fewer safety issues from immune responses, numerous preclinical and clinical studies have expanded [9,33,34,35,36], and more than 400 clinical trials have been registered according to clinicaltrials.gov since the first MSC clinical trial in 1995 [37].

However, the efficacy of MSCs in animal studies has not been fully translated into clinical practice [38,39]. Several reasons can be considered for these discrepancies. First, the process related to MSC preparation can differ between studies. MSCs are known to be widely distributed among various organs and tissues. Manufacturing processes related to the isolation of MSCs from different sources, culture protocols, expansion levels and status of the cells can affect MSC therapeutic efficacy. Second, the donor-recipient match could also be a reason for the observed discrepancies. MSCs in clinical trials are allogenic or autologous, while MSCs used in preclinical studies are applied with xenografts. Disease conditions that include immune systems between humans and other animals can result in nonidentical effects. Finally, cell delivery route and dose can affect the efficacy of transplantation. For instance, blood vessels are an effective route for drug administration in most organs because of their accessibility throughout the body but dosing through blood vessels is less effective in delivering cells to the brain due to the blood–brain barrier. Instead, thecal administration can be a potential delivery route to the central nervous system, although the concentration may influence the distribution of the cells in the brain [40].

A close correlation between the therapeutic efficacy of MSCs and the secreted biomolecules, including extracellular vesicles, is widely known [41]. Therefore, elucidating the composition and functions of secreted proteins from MSCs is critical, but differences can exist among their various origins. Several comparative analysis studies of secretomes according to the origins of MSCs have been published recently. A study by Mead and colleagues demonstrated that dental pulp MSCs are the most advantageous choice for neuroprotection and neuritogenesis when compared with MSCs from BM and AD [42]. Pires and colleagues revealed that the secretome of MSCs from BM might be optimal for reducing oxidative stress, while the conditioned media of cord and AD-MSCs are more beneficial for reducing excitotoxicity [43]. Talwadekar and coworkers showed that the secretome of PL-MSCs was a better option for immune regulation compared to cord tissue of the same donor [44]. Wolbank and colleagues found dose-dependent immunomodulatory effects in human amniotic stem cells and AD-MSCs [45]. However, these studies focused on certain functional features of the secretome, such as neuroprotection and immunomodulation, which may limit the scope of application to various diseases. A study from Tachida and colleagues reported the secretome profile of MSCs from BM, AD, and dental pulp, yet it is difficult to apply these results clinically since their results were acquired from rat MSCs [46]. To consider the optimal MSC source for clinical application, secretome profiling, and comparison studies without bias for specific diseases need to be conducted with human MSCs.

In this study, we characterized and compared the secretomes of AD, BM, PL, and WJ-MSCs identified by mass spectrometry. Common functions of the secretome from all four MSCs indicated that they provide a trophic cellular niche in general, although the protein profiles vary among the sources. All groups are expected to increase the migration and invasion of cells and were also predicted to increase the survival of cells by reducing apoptosis and necrosis. Common biological properties in the secretomes from the four different sources were related to ECM organization, cellular homeostasis and relevant signaling pathways, although activation levels differed among sources. Rearranging a niche and inducing the regeneration of nearby cells is closely related to the therapeutic potential of MSCs. Secreted proteins from all four types of MSC were also related to migration, cellular developmental and metabolic processes, although the secretome from fetal MSCs was more correlated than adult MSCs. There is a chance that these features may be due to birth-related origins; however, further research is needed to elucidate the differences.

Interestingly, despite their different profiles, the functional analyses of source-specific secretomes were related to promoting cellular development, cell proliferation and anti-apoptosis. [7]. Biological pathways were predicted not by the common proteins of WJ and PL-MSCs, but by the proteins that differed between them and were associated with cell proliferation and cell migration (Figure 5d). Proteins related to the migration of cells were enriched in the secretome of all MSCs. MSCs are known to recruit endogenous cells to repair lesions and regulate immune responses when transplanted [47,48,49,50,51,52,53,54,55,56], and their clinical safety is well reported [38,57,58], though their usage should be undertaken with caution in patients with a history of cancer [59,60,61], as it is an excluding criterion for clinical trials evaluating MSC transplantation. It was also found that proteins related to vasculogenesis and angiogenesis are upregulated. It seems that different protein expressions can lead to the same physiological functions through various pathways [62,63,64]. These results suggest that the regenerative capacity of MSCs mainly depends on their secretome [63,65,66].

In addition to the five major groups mentioned in the results, there were also protein groups that were increased in BM and WJ-MSCs and increased in BM and PL-MSCs. However, the number of proteins in these two groups was as low as 10–20 and did not show significant enrichment of any biological function.

Our study has several limitations to be discussed. First, although we have identified the secretome differences in MSCs from four different sources, microenvironmental cues in different diseases may result in different secretion patterns [33]. The features of MSCs the regulate homeostasis in the tissue environment are well-known. In this respect, the source-specific secretome that we have identified can be expected to have the unique properties of its origin. However, for the therapeutic application of MSCs, further research is required to uncover the difference between MSC secretomes from various sources by disease condition. Second, the secretome of MSCs in normal culture and serum-free conditions can be different as serum-free medium (SFM) can result in a lack of nutrients. The secretome collection procedure was performed to minimize the interference between the functions of the secretome in normal and SFM conditions [67], and the protein lists from bovine serum were excluded. Since the use of fetal bovine serum (FBS) is strongly suggested to be excluded in the clinical use of MSCs, an analysis of characteristics in conditioned SFM could be meaningful.

We have identified and analyzed the secretomes of MSCs from four different sources by LC–MS/MS. Further analysis showed distinct proteome profiles depending on the tissues, though they shared functional features, which can be represented as cell proliferation, cell migration, and anti-apoptosis. When compared with adult MSCs, fetal MSCs secreted more diverse proteins, thus exhibiting stronger functional properties and relevance. Various proteins that were not identified in the AM or BM-MSC secretomes but only in the PL and WJ-MSC secretomes seem to have a great influence on function prediction. The secretome pattern may change in a disease environment, but our results show that fetal MSCs might have higher therapeutic potential. Our findings may be beneficial in further research to validate the clinical application of MSCs in various diseases.

## 4. Materials and Methods

### 4.1. Isolation and Culture of MSCs from four Different Sources

Human BM-MSCs were kindly provided by Prof. Dong Ik Kim’s laboratory at the Samsung Medical Center. Human MSCs were isolated from three different sources, AD, PL and WJ. Lipoaspirated tissue was collected in a 50 mL conical tube and weighed. The AD tissue was washed extensively with phosphate-buffered saline (PBS) and minced. An equal volume of Dulbecco’s PBS (DPBS; Gibco, Waltham, MA, USA) with 0.075% collagenase type 1 (Sigma-Aldrich, Saint Louis, MO, USA) was added to break down the ECM and shaken at 200 rpm for an hour at 37 °C. The same volume of minimum essential media α (MEMα; Gibco) with FBS(Gibco) was then added for the inactivation of the enzyme, and the resulting solution was filtered with 100 μm cell strainer and centrifuged at 400× *g* for 5 min. The supernatant was discarded, and the cells were plated in a 75-T flask.

PL tissue was washed with DPBS without calcium or magnesium (Gibco) to remove blood. The chorionic tissue was separated from the amnion, and the basal portion of the chorionic trophoblast layer was minced mechanically. Samples were then digested with 0.2% collagenase type II (Sigma-Aldrich) at 37 °C for 2–3 h. Digested tissues were passed through a 70 μm cell strainer and subsequently centrifuged at 1000× *g* for 5 min and plated in a 75-T flask.

To isolate cells from WJ, the umbilical cord was washed to remove blood and blood clots and cut into 1.5 cm length pieces. Each piece was then cut longitudinally, and umbilical arteries, veins and umbilical cord adventitia were removed to obtain WJ. The remaining gelatinous tissue surrounding the vessels was collected and minced into fine pieces, placed in sterile 50 mL centrifuge tubes in 0.2% collagenase type I solution. After 40 min, an equal volume of MEMα with FBS was added, and a 70 μm cell strainer was used for the filtration of the tissue sample. Filtered samples were centrifuged at 400× *g* for 10 min, the supernatant was discarded, and the cells were plated in a 75-T flask.

The four types of MSCs from each different source were isolated and expanded in MEMα media containing 10% FBS and 50 μg/mL gentamicin (Gibco) at 37 °C with 5% CO_2_ in an incubator. Passage 6 MSCs were used in this study.

Ethical approval was given by the Institutional Review Board of the Samsung Medical Center, and the four different sources were collected with informed consent obtained from healthy donors (SMC IRB file No. 2016-07-102).

### 4.2. Stemness Evaluation of MSCs

For the evaluation of differentiation potential, tri-lineage differentiation was performed. For osteogenic and adipogenic differentiation, cells were incubated in a StemPro differentiation kit (Gibco) according to the manufacturer’s instructions. Differentiation medium was replaced every 3 days, for 3 weeks. Differentiated cells were fixed with 4% paraformaldehyde (PFA; Sigma-Aldrich), and the respective immunostaining experiments were performed using the following staining solutions: osteogenic, Alizarin Red S (Sigma-Aldrich) and adipogenic, oil red O (Sigma-Aldrich). For chondrogenic differentiation, 2 × 10^5^ cells were pelleted in 15 mL conical tubes. The cell pellets were suspended in 500 μL of chondrogenic differentiation medium with high-glucose Dulbecco’s modified Eagle’s medium (DMEM; Gibco) supplemented with 10 ng/mL of recombinant human transforming growth factor-β3 (TGF- β3; R&D Systems, McKinley Place, NE, USA), 500 ng/mL of bone morphogenic protein-6 (BMP-6; R&D Systems, McKinley Place, NE, USA), 0.6 μg/mL of dexamethasone (Sigma-Aldrich), 1% insulin–transferrin–sodium selenite solution (ITS; insulin 25 μg/mL, transferrin 25 μg/mL, and sodium selenite 25 ng/mL; Sigma-Aldrich), 40 μg/mL of l-proline (Sigma-Aldrich), 50 μg/mL of ascorbic acid-2-phosphate (Sigma-Aldrich), and 100 μg/mL of sodium pyruvate (Sigma-Aldrich). Differentiation medium was replaced every 3 days, for 4 weeks. Chondrocytes were fixed with 4% PFA, dehydrated by using ethanol and embedded in OCT compound (Scigen, Paramount, CA, USA). Blocks were sectioned at 5 μm thickness using a cryotome (Thermo Fisher Scientific, Waltham, MA, USA), and the sections were stained with safranin-O (Sigma-Aldrich). Stained slides were observed using an inverted light microscope (CKX41; Olympus, Shinjuku-ku, Tokyo, Japan).

To confirm the stemness of MSCs, cell surface marker analysis and mesodermal differentiation assays were performed. Harvested MSCs were blocked with PBS supplemented with 2% FBS. Immunophenotypic characteristics of MSCs were examined using flow cytometry for the following markers: CD73, CD90, CD105, CD11b, CD19, CD34, CD45 and HLA-DR (MHCII; BD Biosciences, San Jose, CA, USA). At least 10,000 events were acquired by using a BD FACS Verse flow cytometer, and the results were analyzed by using the BD FACSuite software version 10.

### 4.3. Preparation of the Secretome

MSCs were cultured until 70% confluence, washed three times with SFM omitting phenol red, and incubated in that the same SFM for an additional 24 h. The protease inhibitors phenylmethylsulfonyl fluoride (Sigma-Aldrich) and ethylenediaminetetraacetic acid (USB, Cleveland, OH, USA) were added to the conditioned medium at final concentrations of 2 mM and 1 mM, respectively. Cell debris was removed by centrifugation (400× *g*, 20 min, 4 °C) and sterile filtration (pore size: 0.22 μm, Millipore, Billerica, MA, USA). The medium was concentrated by ultrafiltration using an Amicon Ultra-15 centrifugal filter device (nominal molecular weight limit, 10 kDa, Millipore) and washed three times with 8 M urea, 75 mM NaCl and 50 mM Tris, pH 8.2 [68].

### 4.4. In-Solution Digestion

The collected secretome samples (100 μg protein) were reduced with 5 mM dithiothreitol (Thermo Fisher Scientific) at room temperature for 1 h and alkylated with 15 mM iodoacetamide (Sigma-Aldrich) at room temperature for 2 h in the dark. The samples were diluted 10-fold with 50 mM Tris and 75 mM NaCl (pH 8.0) to reduce the urea concentration in the sample to less than 1 M. The secretome was digested with sequencing grade modified trypsin (Promega, Madison, WI, USA) at 37 °C for 16 h with vigorous shaking. The ratio of enzyme to protein was 1:50. The protein digestion reaction was stopped by adding trifluoroacetic acid to 0.1% final concentration. The protein digests were desalted using an Oasis HLB 1 cc Vac cartridge (Waters, Milford, MA, USA). Before loading the samples, the cartridges were sequentially washed with 1 mL of 50% acetonitrile in water and three times with 1 mL of 0.1% formic acid and 5% acetonitrile in water. After loading the digested sample, the cartridges were washed three times with 1 mL of 0.1% formic acid and with 5% acetonitrile in water. The peptides were eluted into a clean tube with 800 μL of 0.1% formic acid, 40% acetonitrile in water and 200 μL of 0.1% formic acid, 80% acetonitrile in water, dried in vacuo, and stored at −80 °C until use.

### 4.5. Liquid Chromatography and Tandem Mass Spectrometry (LC–MS/MS)

The peptide samples were reconstituted in 0.4% acetic acid and sonicated in a sonication bath at 35 °C for 10 min. An LTQ-Orbitrap XL mass spectrometer (Thermo Fisher Scientific) was used, and 0.5 µg of the sample was injected into a reversed-phase C18 column (20 cm × 75 μm i.d., 3 μm, 120 Å, packed in-house; Dr. Maisch, Beim Brückle, Ammerbuch-Entringen, Germany) on an Eksigent NanoLC-ultra 1D plus system at a flow rate of 400 μL/min. The column was pre-equilibrated with 95% solvent A (0.1% formic acid in water) and 5% solvent B (0.1% formic acid in acetonitrile) for 16 h. The peptides were eluted at a flow rate of 400 μL/min with a linear gradient of 5–40% B for 240 min, followed by 80% B wash at 300 nL/min for 35 min and 5% B re-equilibration at a flow rate of 300 nL/min for 10 min. ESI spray voltage was set to 2.1 kV, capillary voltage to 21 V and the temperature of the heated capillary to 250 °C. MS survey was scanned from 350 to 1800 *m*/*z*, and the top 10 ions were selected for data-dependent MS/MS scans with the following parameters: charge state, ≥2; isolation width, 2 *m*/*z*; normalized collision energy, 35%; dynamic exclusion duration, 60 s. All data were acquired using Xcalibur software v2.2 (Thermo Fisher Scientific).

### 4.6. Analysis of Mass Spectrometric Data

To avoid false identification of residual FBS-derived proteins as human proteins in the process of MS, we used the human UniProtKB database (released in 2016, 12) with an FBS protein list added for MS data search to exclude suspected proteins affected by FBS [69]. The LFQ method was analyzed through the MaxQuant program (v1.5.6.0, Max Planck Institute of Biochemistry, Martinsried, Germany). The first peptide tolerance was selected as 20 ppm, and the main search peptide tolerance was 4.5 ppm. Search parameters were two missed trypsin cleavage sites, cysteine carbamidomethylation (+57.0215 Da) as a fixed modification, methionine oxidation (+15.9949 Da) and N-terminal protein acetylation (+42.0106 Da) as variable modifications. The false discovery rate (FDR) for peptide and protein identification was set to 1%. The minimum number of razor and unique peptides in a protein group was considered as 1 peptide as protein identification. Data processing was performed using the Perseus program (v1.6.0.7, Max Planck Institute of Biochemistry) to validate the proteins that characterize MSCs. For reliable quantification, proteins identified in more than two MSCs from the same source were used. The LFQ intensities were compared between different MSCs using the mean value of samples to select specific proteins. Among two MSC groups, proteins with an intensity higher than 1.5-fold from the other MSC were selected. Mean values were ignored when only one sample was identified in one MSC group.

### 4.7. Bioinformatics Analysis

GO terms were analyzed using the algorithm of the database for annotation, visualization and integrated discovery (DAVID) tools (https://david.ncifcrf.gov/). The cutoff parameter of the FDR for the analyzed GO term was less than 0.01. GO enrichment was performed using Fisher’s exact test in Perseus software. The missing intensity value of unidentified proteins was imputed for clustering.

The analysis tool provided by DTU bioinformatics was used for the secretome analysis. (Department of Bio and Health Informatics, http://www.cbs.dtu.dk/services/). Using the accession number of the identified proteins, we converted the FASTA format file through the retrieve/ID mapping of the UniProt ID. SignalP V4.1 tool and used this to predict classically secreted proteins based on secretion signals. These secretory signals contain transmembrane sequences, and we used the default cutoff value provided by SignalP [70]. We predicted non-classically secreted proteins that were secreted by signal peptide independent secretion pathways using SecretomeP v2.0 [71]. Proteins with more than 4000 amino acids were analyzed using only the N-terminal 4000 amino acids, and those with an NN-score ≥0.5 were selected as non-classically secreted proteins. Lastly, with proteins that were not analyzed in both SignalP and SecretomeP, THMHH v2.0 was used to select any transmembrane proteins. A protein was considered to be a transmembrane protein if it had a sequence with transmembrane helices [72]. Because transmembrane proteins can be present in extracellular vesicles or exosomes of the secretome, we considered all proteins, predicted as either classically secreted, non-classically secreted or membrane proteins, to be truly secreted proteins. The proteins not included in the three prediction results were classified as other proteins. The prediction of biological functions and protein network analysis was performed using the IPA program (Qiagen, Redwood city, CA, USA). A t-test was performed using the LFQ intensity, and proteins with *p*-values less than 0.05 were used for IPA analysis. For biological function prediction, a *p*-value of less than 0.01 was used in the results. Protein networks for biological functions used activation z-scores greater than 1.5.

## Supplementary Materials

The following are available online at https://www.mdpi.com/1422-0067/22/2/845/s1.
