# Supplementary material for: Comparative Proteomic Analysis of the Mesenchymal Stem Cells Secretome from Adipose, Bone Marrow, Placenta and Wharton’s Jelly
Source: Int J Mol Sci. 2021 Jan 15;22(2):845. doi: 10.3390/ijms22020845 (PMC7829982; doi:10.3390/ijms22020845)

**Supplementary Materials**

Supplementary Figure 1: Figure S1. Negative cell surface markers of MSCs CD34, CD45, CD19, and CD11b of mesenchymal stem cells (MSCs) are shown. AD-MSC, adipose-derived MSCs; BM-MSC, bone marrow-derived MSCs; PL-MSC, placenta-derived MSCs; WJ-MSC, Wharton’s jelly-derived MSCs. Supplementary Figure 2: Comparison of identified peptides among three technical replicates and among four types of MSCs. Supplementary Figure 3: Comparison of identified proteins among three technical replicates and among four types of MSCs. Supplementary Figure 4: Predicted networks of proteins involved in the migration of cells from each group. Supplementary Figure 5: Networks of proteins found at higher levels in each secretome of AD and BM-MSC. Supplementary Figure 6: Predicted networks of proteins related to source-specific biological function of PL and WJ-MSC secretomes. Supplementary Figure 7: The biological function of foetal-derived MSCs in the secretome predicted to be at higher levels than in adult-derived MSCs.

Figure S1.


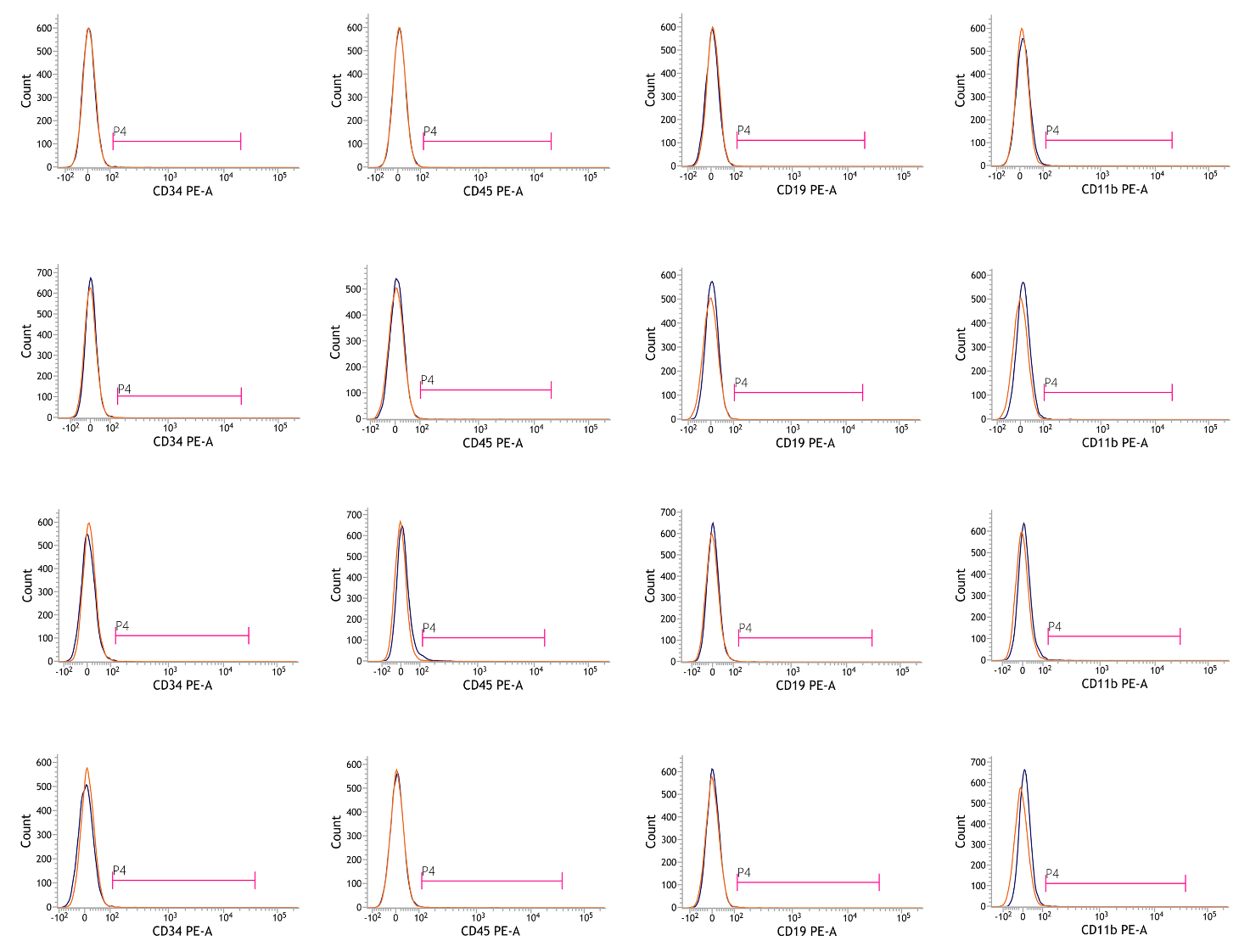


Figure S2.


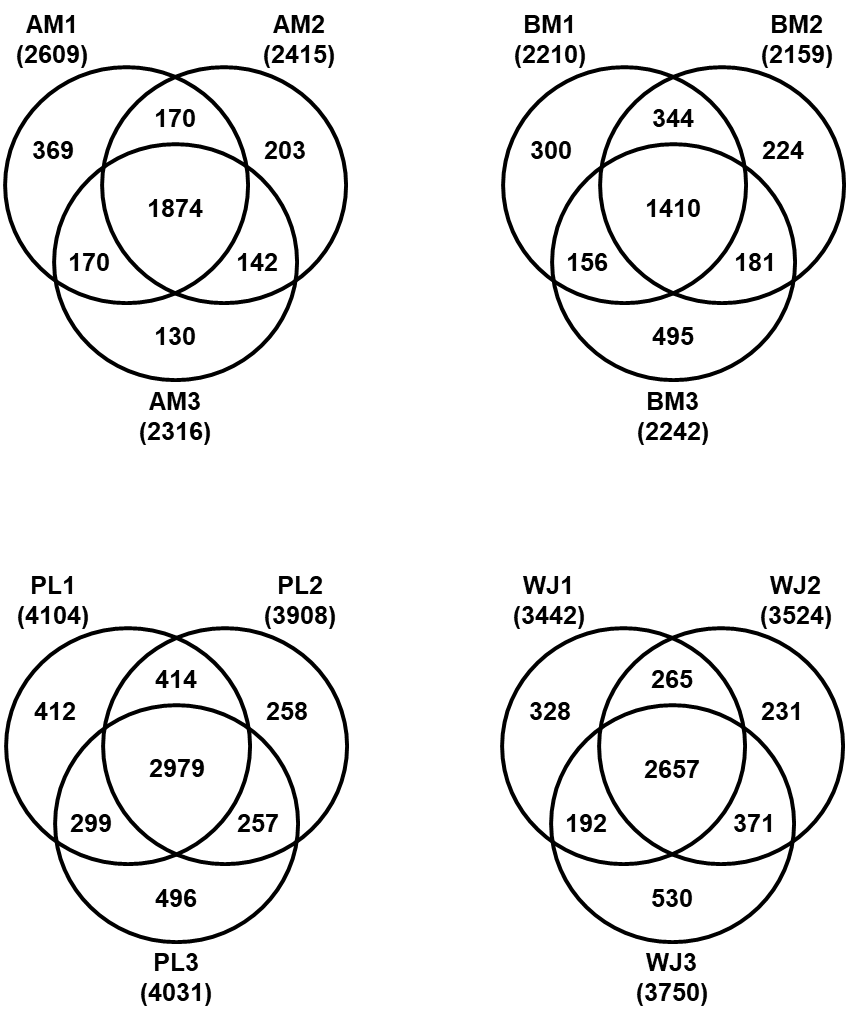


Figure S3.


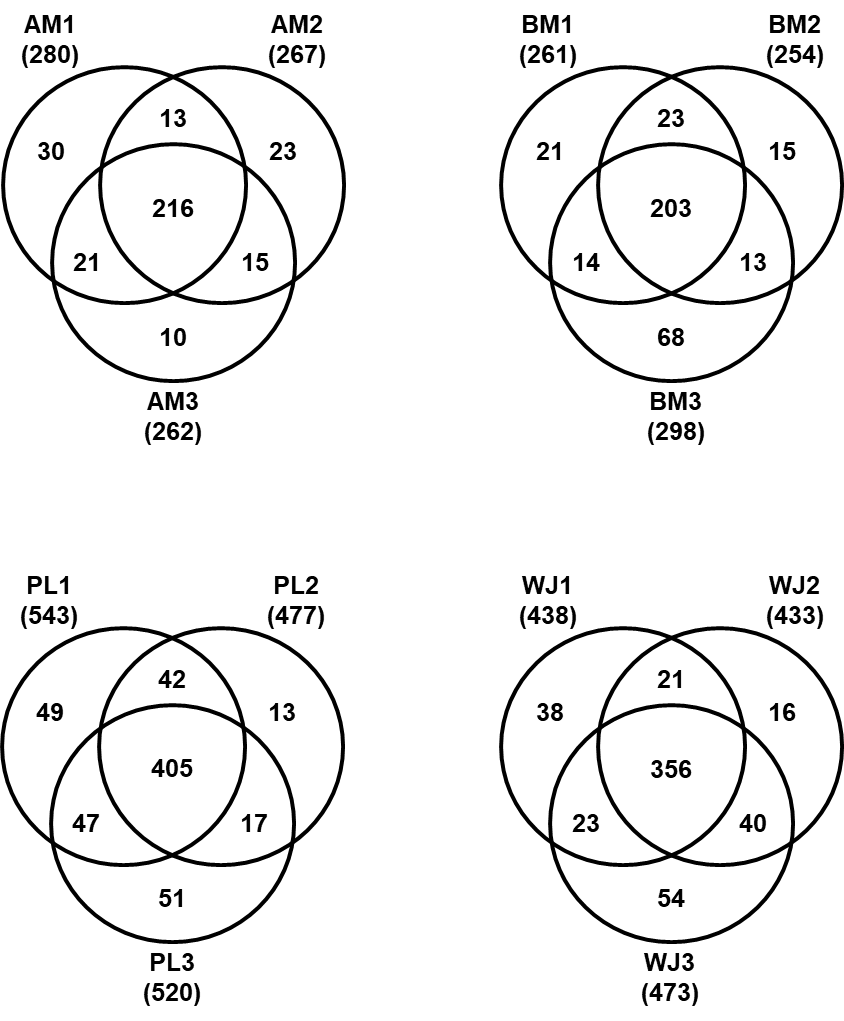


Figure S4.


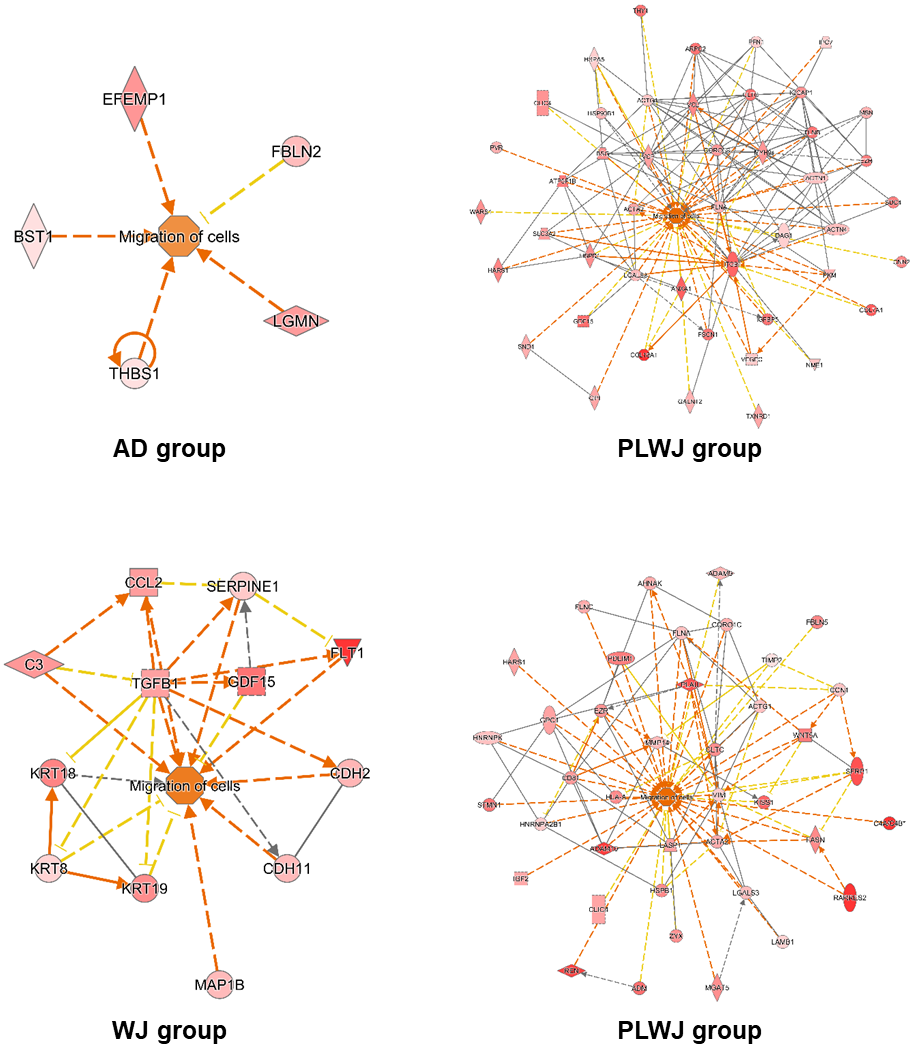


Figure S5.


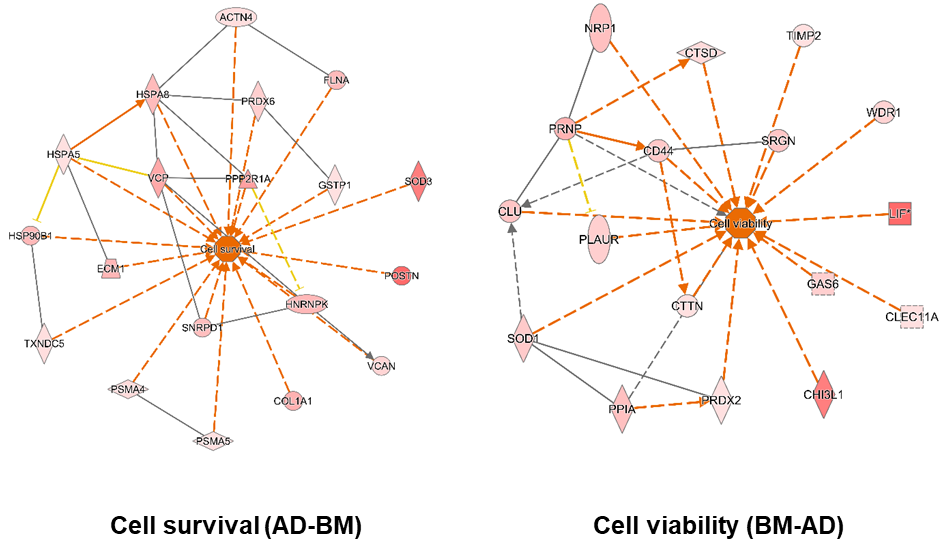


Figure S6.


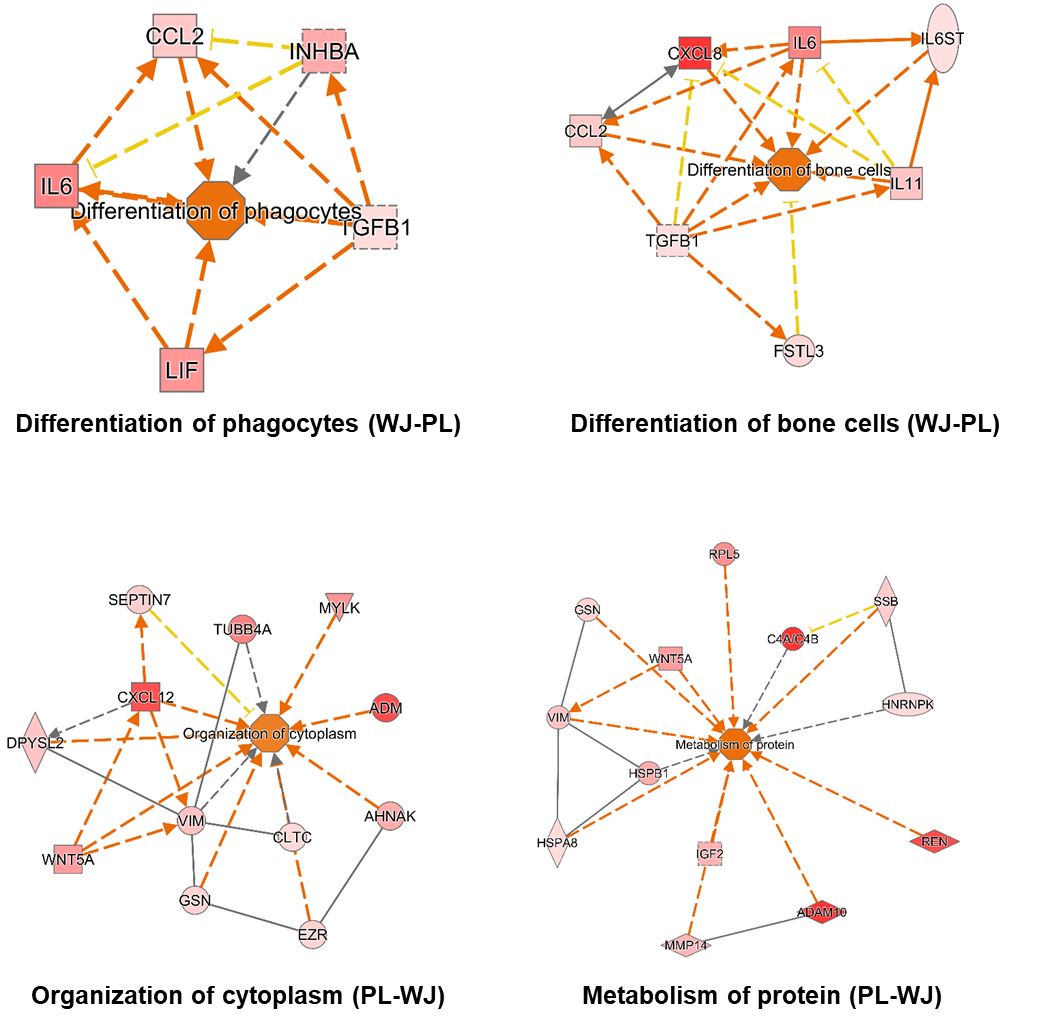


Figure S7.


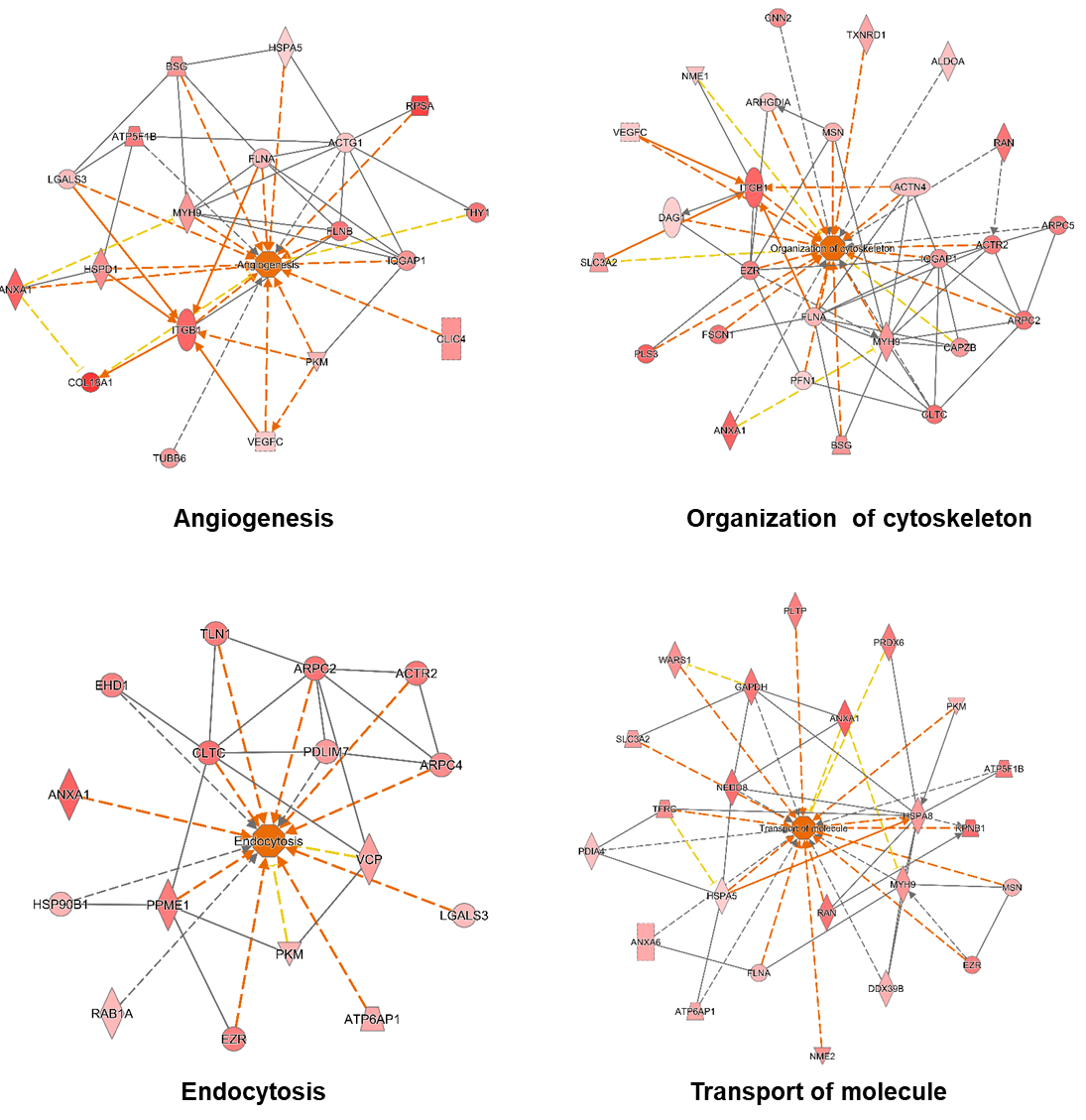

Supplement: Supplementary file 1 [file ijms-22-00845-s001.zip › ijms-1072739-SupplementaryMaterials/Supplementary Figures.docx]
